# Female C57BL/6J Mice Show Alcohol-Seeking Behaviour after Withdrawal from Prolonged Alcohol Consumption in the Social Environment

**DOI:** 10.1093/alcalc/agab032

**Published:** 2021-04-24

**Authors:** Maryna Koskela, T Petteri Piepponen, Jaan-Olle Andressoo, Vootele Võikar, Mikko Airavaara

**Affiliations:** Institute of Biotechnology, HiLIFE, University of Helsinki, PO Box 56, Viikinkaari 5, 00014, Helsinki, Finland; Neuroscience Center, HiLIFE, University of Helsinki, PO Box 56, Biomedicum, Haartmaninkatu 8, 00014, Helsinki, Finland; Division of Pharmacology and Pharmacotherapy, Faculty of Pharmacy, University of Helsinki, PO Box 56, Viikinkaari 5, 00014, Helsinki, Finland; Department of Translational Neuroscience, Faculty of Medicine, University of Helsinki, PO Box 56, Biomedicum, Haartmaninkatu 8, 00014, Helsinki, Finland; Neuroscience Center, HiLIFE, University of Helsinki, PO Box 56, Biomedicum, Haartmaninkatu 8, 00014, Helsinki, Finland; Institute of Biotechnology, HiLIFE, University of Helsinki, PO Box 56, Viikinkaari 5, 00014, Helsinki, Finland; Division of Pharmacology and Pharmacotherapy, Faculty of Pharmacy, University of Helsinki, PO Box 56, Viikinkaari 5, 00014, Helsinki, Finland; Neuroscience Center, HiLIFE, University of Helsinki, PO Box 56, Biomedicum, Haartmaninkatu 8, 00014, Helsinki, Finland

## Abstract

**Aims:**

Recently we developed a model to study alcohol-seeking behaviour after withdrawal in a social context in female mice. The model raised several questions that we were eager to address to improve methodology.

**Methods:**

In our model, female mice were group-housed in automated cages with three conditioned (CS+) corners and water in both sides of one separate non-conditioned corner. Water was available with opened doors at all the time of training. We established conditioning by pairing alcohol drinking with light cues. Here, we introduced prolonged access to increasing concentrations of alcohol instead of intermittent access. To study motivation to drink alcohol, we carried out the extinction tests on withdrawal days 1 (WD1) and 10 (WD10). During tests, the light cues were present in conditioned corners, but there was no liquid in the bottles.

**Results:**

We found that the number of visits and nosepokes in the CS+ corner in the alcohol group was much higher than in the water group. Also, during training, the consumption of alcohol was increasing. In the extinction tests, we found that the number of nosepokes in the CS+ corner increased in the alcohol group on both WD1 and WD10.

**Conclusions:**

Our study supports that alcohol-seeking behaviour after withdrawal can be modelled and studied in group-housed animals and environments without social isolation.

## Teaser

**Short Summary:** We developed a model to study alcohol drinking behaviour in an enriched and social environment. Long-term conditioning coupling with alcohol reward results in cue-induced alcohol-seeking behaviour in group-housed female C57BL/6J mice. Moreover, a high number of nosepokes on the last day of alcohol drinking conditioning might potentiate alcohol-seeking after withdrawal response.

## INTRODUCTION

A major challenge for treating drug addiction is a relapse that occurs after a long abstinence period. More than 30% of alcohol addicts experience relapse after 3 years of abstinence (Hunt *et al.*, 1971; Bottlender and Soyka, 2005). Intense drug craving, uncontrollable need or desire of a drug, can be evoked by drug-associated stimuli (cues), contexts or stressors (Bossert *et al*., 2013). A critical factor in craving is associative learning when drug consumption is paired with conditional stimuli (Weiss, 2005). In humans, craving can occur in response to environmental stimuli that bring memories about the pleasurable effect of drug-taking. Moreover, it has been reported that cue-induced craving in people with alcohol use disorder is higher after 60 days of abstinence compared with 7 days (Li *et al*., 2015). Here, we would like to emphasize that craving is complex set of experiences in behaviour reported explicitly by humans.

To date, animal models in addiction research are critical tools to study mechanisms underlying different aspects of the disease progression (Becker and Ron, 2014). Recently we have reported a model where one of the aspects of alcohol craving, alcohol-seeking after withdrawal, can be studied in group-housed female mice in automated cages (Koskela *et al*., 2018). The model allows studying cue-associated alcohol-seeking after withdrawal without human interference and causing stress to animals. Our results of the cue-induced extinction tests after alcohol withdrawal suggested that conditioned cue coupled with long-term intermittent alcohol drinking is the crucial factor in the development of cue-induced alcohol-seeking behaviour. However, the presentation of the model raised discussion regarding possible confounding factors. Thus, we modified our protocol to address the issue.

Here, we extended the alcohol drinking conditioning period. Alcohol was available on both sides in three corners. The nosepoke on either side led to light-cue. Water was freely available on both sides of one corner, and the doors were open to both sides all the time. Mice had access to water without a nosepoke-induced door opening. These changes allowed us to eliminate the following possible confounding factors: (a) the door opening to water bottles after the nosepoke; (b) CS+ and CS− sides in the same corner and (c) extinction during non-alcohol day. This study supports our previous findings that coupling alcohol drinking with light cues is a crucial factor in developing alcohol-seeking behaviour after withdrawal in female group-housed mice.

## MATERIALS AND METHODS

### Ethics statement

The animal experiments were performed according to the EU legislation harmonized with Finnish legislation and have been approved by the National Animal Experiment board of Finland (ESAVI/7812/04.10.07/2015, ESAVI/19348/2019).

### Experimental animals

The behavioural experiments were performed in female C57BL/6JRccHsd mice (*n* = 48, Envigo). The mice arrived at the age of 8 weeks old and were housed under temperature-controlled conditions at 20–22°C in a 12-h light/dark cycle with lights on at 06.00 am with *ad libitum* access to standard lab chow and water. The mice were individually recognized by radio-frequency identification (RFID) transponders (Planet ID GmbH, Germany). The transponders were implanted under the skin under 2.5% isoflurane anaesthesia 1-week before experiments began. The animals were 10 weeks old, average 19 g of weight and grouped eight mice per cage at the beginning of the experiments. There were 24 animals in the alcohol group and 24 animals in the control water group.

**Fig. 1.**
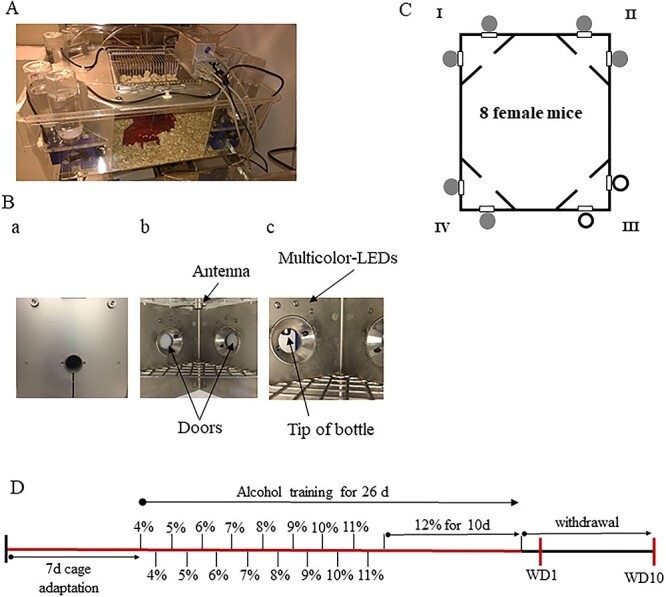
Experimental design. (**A**) Picture of an automated cage IntelliCage. (**B**) Detailed picture of the corner of the IntelliCage: (**a**) entrance to the corner; (**b**) two sides of the corner with closed automated doors and RFID reader (antenna); (**c**) side of the corner with opened automated door. (**C**) Schematic representation of the experimental settings in the automated cage used in this study. The corners of the cage are marked in Roman numerals. Sides with conditional stimulus are coloured in grey whereas non-conditional sides are coloured in white. (**D**) Representation of the experimental timeline. Red lines represent the time spent in the automated cages.

Behavioural analysis was done using an automated IntelliCage system, as shown in Fig. 1A (TSE, Bad Homburg, Germany) (Koskela *et al*., 2017; Koskela *et al*., 2018). The automated cage allows performing experiments without handling the mice under fully automated conditions in the home cage environment (Fig. 1A). The cages were controlled by a computer with IntelliCage Plus software, performing pre-programmed experimental schedules.

The mouse enters the corner through the hole (Fig. 1B,a). All the cage corners have an antenna that reads RFID signals and two sides with doors (Fig. 1B,b). When the door is open, the mouse can lick the tip of the bottle (Fig. 1B,c). Every side has a multicolour-LEDs (Fig. 1B,c).

During the experiment, the following behavioural parameters were recorded: number of visits to the corner, number of nosepokes to the door and number of licks. The schematic representation of the cages during experiments is shown in Fig. 1C. A green light was used as a conditional stimulus (CS+). Four triangular red shelters (Tecniplast, Buguggiate, Italy) were placed in the middle of the cages. They were used as sleeping quarters and as a stand to reach the food. The floor of the cage was covered with a layer of bedding.

### Behavioural procedure

The experimental timeline is presented in Fig. 1D. Totally six cages were used simultaneously during the experiment: three cages for alcohol and three for the control water group of mice. The mice were randomly placed in automated cages in groups of eight animals per cage. The first week was the habituation period. It consisted of the free adaptation phase (3 days, all doors in all corners were open, animals could enter and drink water in any corner) and the nosepoke adaptation phase (4 days, all doors in all corners were closed, nosepoke opened the door for 7 s). The adaptation period is required for the animal to learn to enter the corner and drink there (Voikar and Gaburro, 2020). After that, in cages with alcohol group of mice, alcohol was placed in three corners (I, II and IV) and water was available in one corner (III). Nosepoke opened the door in the alcohol corner for 7 s and switches on the green LED light until the end of drinking. The schematic set up in the automated cage is presented in Fig. 1B. Water in the water corner was available with opened doors at all the experiment time. However, the number of nosepokes was recorded. The control water group had water in all corners and the same green light settings in corners I, II and IV, and open doors in corner III.

In cages with alcohol group of mice, the bottles with ethanol were switched every third day between 10.00 and 11.00 am. First, mice were exposed to 4% (v/v) ethanol solution for 2 days. We increased ethanol concentration by 1% every 2-day until 12% ethanol solution was reached. After that, the mice continued the training period for 10 days with access to 12% ethanol in alcohol corners.

There were two times during the training period when we introduced cage cleaning breaks. We did the first and the second cage cleanings after mice had spent 10–11 days in cages, namely, on days when 6 and 12% alcohol solutions were introduced. However, there was no cleaning after 10 days of 12% alcohol drinking to reduce novelty-induced exploratory activity during the extinction tests.

### Alcohol withdrawal phase

After the training period, we removed the mice from automated cages to standard home cages. For the extinction tests, we brought the mice to the automated cages and, after each test, returned to the standard home cages.

### Extinction tests in automated cages

The extinction tests were performed on Days 1 and 10 after the training period between 10.00 and 11.00 am. During 1-h tests, the experimental design was similar to the training period, except there was no liquid in the bottles. The bedding material has not been changed after training and has been kept the same for the next 10 days.

### Statistical analysis

Graphs were made and statistical analyses were performed with GraphPad Prism 7 (GraphPad Software, CA, USA). Two-way repeated measure analysis of variance was used and statistically significant effects were followed by Bonferroni’s post-hoc test. The values are given as mean ± SEM and considered to be significant at *P* < 0.05.

## RESULTS

### Alcohol drinking training

**Fig. 2.**
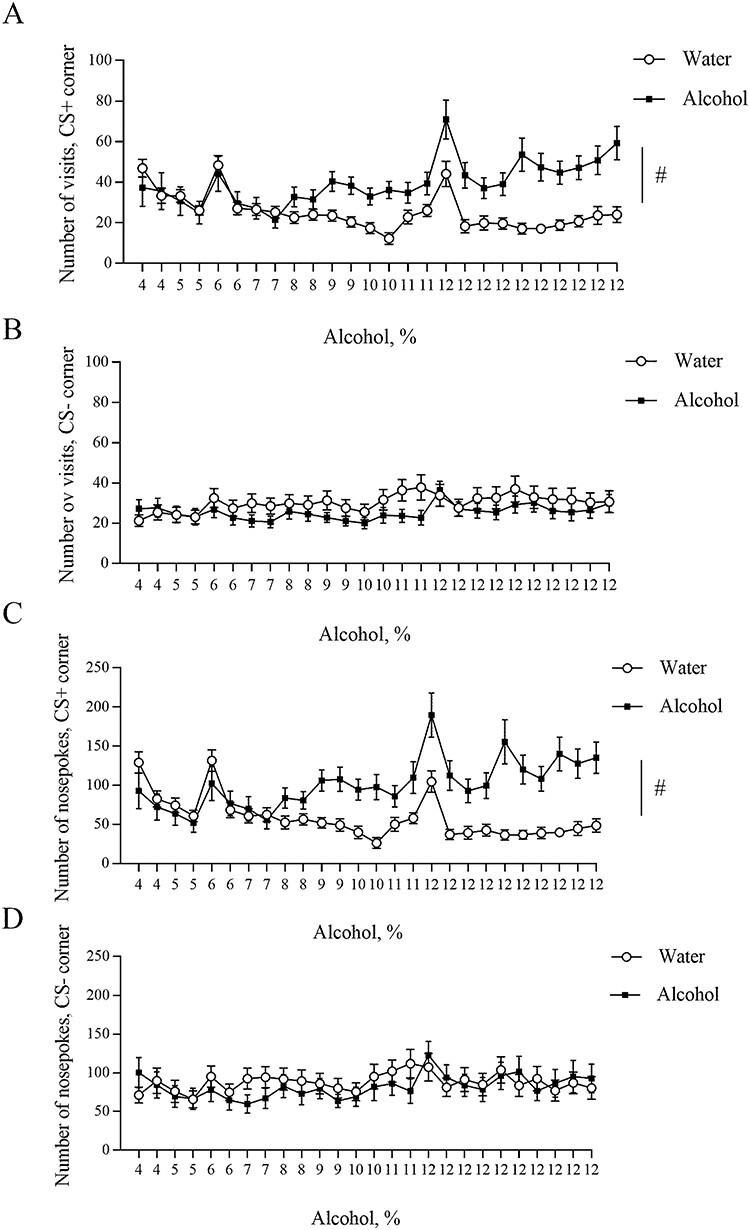
Behavioural activity in the automated cages during training period. (**A**) Number of visits in conditioned corner. (**B**) Number of visits in non-conditioned corner. (**C**) Number of nosepokes in conditioned corner. (**D**) Number of nosepokes in non-conditioned corner. ^#^*P* < 0.05. All means are presented with their standard errors (± SEM).

Analysis of the behavioural activity during the training period revealed that the number of visits was different on different training days (Fig. 2A, Day effect, *F*(25, 575) = 11.05, *P* < 0.0001). Also, the number of visits differs significantly between alcohol and water groups during the training (Day x Training Drug interaction, *F*(25, 575) = 7.87, *P* < 0.0001). The between-subjects analysis showed a significant difference in the number of visits in the conditioned corner between alcohol and water groups (*F*(1, 23) = 5.625, *P* = 0.0265). In contrast, the number of visits in the non-conditioned corner was similar for both groups (Fig. 2B, *F*(1, 23) = 0.6483, *P* = 0.4289). In our previous study, we could not determine whether there is a difference in the number of visits because both conditional and non-conditional sides were in one corner (Koskela *et al*., 2018).

**Fig. 3.**
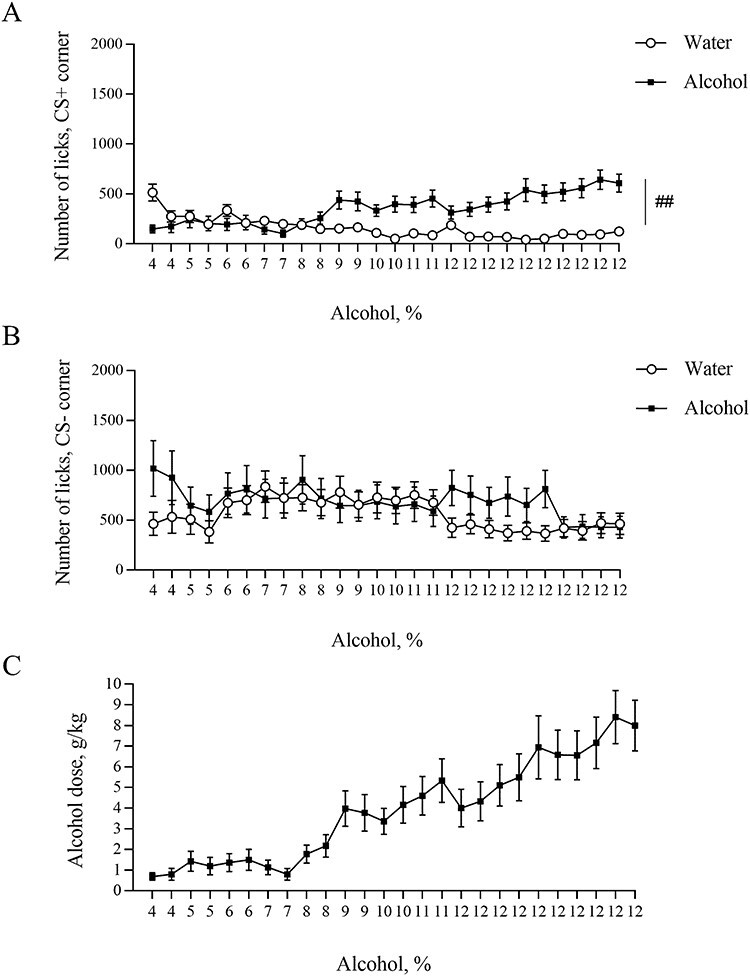
Number of licks in the automated cages during training period. (**A**) Number of licks in conditioned corner. (**B**) Number of licks in non-conditioned corner. (**C**) The ethanol dose that mice consumed during alcohol training period was estimated as g/kg/24 h. ^##^*P* < 0.01. All means are presented with their standard errors (± SEM).

The within-subjects effects analysis for the number of nosepokes during the training showed a significant increase in the number of nosepokes (Fig. 2C, Day effect, *F*(25, 575) = 10.25, *P* < 0.001). Moreover, the within-subjects effects demonstrated a significance for Day x Training Drug interaction (*F*(25, 575) = 10.03, *P* < 0.001), showing that the number of nosepokes differs during the training between alcohol and water groups. Furthermore, between-subjects analysis showed that there is a significant difference in the number of nosepokes in conditioned corner between alcohol and water group (*F*(1, 23) = 7.418, *P* = 0.0121), whereas there was no difference in non-conditioned corner (Fig. 2D, *F*(1, 23) = 0.09714, *P* = 0.7581).

Moreover, we then analysed the visits with nosepokes in rewarded corners. The between-subject analysis showed a significant Training Drug effect, indicating that the alcohol group preferentially visited cue-induced corner as measured by the number of visits with nosepokes. (Supplementary Fig. 1, *F*(1, 23) = 6.302, *P* = 0.0195).

Next, we have analysed the number of licks in conditioned and non-conditioned corners. The within-subjects effects analysis demonstrated a significant training day effect in the conditioned corner as well as Day x Training Drug interaction (Fig. 3A, *F*(25, 575) = 3.356, *P* < 0.0001; *F*(25, 575) = 12.82, *P* < 0.0001 respectively) indicating that the number of licks is different on different days of training. The between-subjects analysis showed a significant difference between alcohol and water groups (*F*(1, 23) = 12.81, *P* = 0.0016), pointing out that the alcohol group of mice performed more licks than the water group.

We then estimated the dose of consumed alcohol-based as previously described (Koskela *et al*., 2018) and found that ethanol dose started from 0.7 ± 0.2 g/kg and peaked at 5.3 ± 1.1 g/kg before 12% ethanol was given (Fig. 3C). After mice got 12% ethanol continuously, the consumed amount increased to 8.4 ± 1.3 g/kg. Interestingly, despite the lower consumption of low concentrated ethanol than our previous results (Koskela *et al*., 2018), the consumption of 12% alcohol was almost similar.

There was an apparent increase in activity during training days when we introduced 6 and 12% alcohol in both alcohol and water groups. At both time points, we cleaned cages by renewing the bedding material in all cages that presumably resulted in an increased natural exploratory activity.

### Extinction tests after alcohol drinking training

**Fig. 4.**
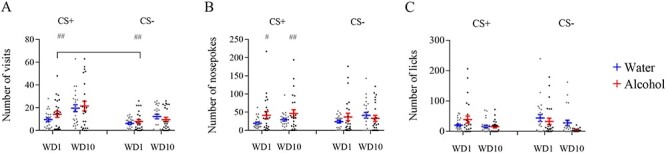
Behavioural activity in the automated cage during extinction tests after training period. (**A**) Number of visits to conditioned and non-conditioned corner in WD1 and WD10. (**B**) Number of nosepokes in conditioned and non-conditioned corners on WD1 and WD10. (**C**) Number of licks in conditioned and non-conditioned corner on WD1 and WD10. ^#^*P* < 0.05, ^##^*P* < 0.01. All means are presented with their standard errors (± SEM).

We transferred mice to standard home cages after training. We then performed the cue-induced extinction tests on withdrawal days 1 (WD1) and 10 (WD10) in automated cages for 1 h. The number of visits was similar between alcohol and water groups in CS+ and CS− corners. However, the within-subject effect analysis showed that there was a significant Day effect (Fig. 4A, *F*(1, 23) = 44.61, *P* < 0.0001). Post-hoc analysis showed that alcohol group mice visited CS+ corner significantly more than CS− on WD1 (*P* = 0.0074, Bonferroni’s test) and WD10 (*P* < 0.0001, Bonferroni’s test).

Next, we analysed the number of nosepokes (a measure of how much mice want alcohol) in CS+ and CS− corners on WD1 and WD10. The between-subjects effects showed a significant Day effect x Training Drug effect interaction (Fig. 4B, *F*(3, 69) = 4.232, *P* = 0.0083), indicating that the number of nosepokes is higher in the alcohol group. Furthermore, the post-hoc comparisons showed that the alcohol group performed a significantly higher number of nosepokes on WD1 and WD10 in the cue-induced corner than the water group (*P* = 0.0046 and *P* = 0.0418, respectively, Bonferroni’s test). We found no statistically significant differences in the non-conditioned corner. The analysis of the between-subjects effects in CS+ and CS− corners for licks showed significant Day effect (Fig. 4C, *F*(3, 69) = 5.011, *P* = 0.0033) as well as significant Day x Training Drug interaction (*F*(3, 69) = 3.555, *P* = 0.0187).

We then analysed an individual number of nosepokes on WD1 and WD10 and classified animals into three groups according to the changes in numbers of nosepokes. The difference in the number of nosepokes in the ‘no changes’ group was no more or less than five nosepokes. In contrast, the difference in the number of nosepokes performed on WD1 and WD10 in the ‘decrease’ and ‘increase’ groups was more than five nosepokes. The analysis showed that 38% of mice from the alcohol group (Supplementary Fig. 2A) and 29% of mice from the water group (Supplementary Fig. 2B) performed a similar number of nosepokes on both WD. We recorded a decreased number of nosepokes on WD10 for 29% of mice from the alcohol group (Supplementary Figure 2A) and 21% of mice from the water group (Supplementary Figure 2B). Interestingly, we found that ~33% of mice from the alcohol group (Supplementary Figure 2A) and 50% of mice from the water group (Supplementary Figure 2B) performed more nosepokes in conditioned corners on WD10 than on WD1. However, the magnitude of the cue-induced nosepokes is higher in the alcohol group than the water group, suggesting that the rewarding drug is required for a profound cue-induced response.

**Fig. 5.**
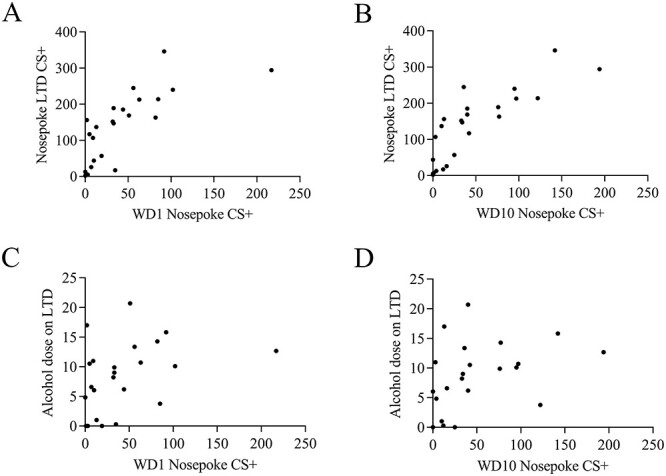
The Pearson correlation coefficient between number of nosepokes on WD1 and WD10 and number of nosepokes on last training day in conditioned corner or consumed alcohol dose on the last day of training period.

### Pearson correlation coefficient

Next, we examined whether the number of nosepokes in the extinction tests on WD1 and WD10 correlates with the number of nosepokes on the last day of alcohol drinking training or the amount of consumed alcohol. The Pearson correlation coefficient analysis showed that there was a high positive correlation between the number of nosepokes on the last training day correlated and the number of nosepokes on WD1 (Fig. 5A, *r* = 0.75, *P* < 0.0001) and WD10 (Fig. 5B, *r* = 0.83, *P* < 0.0001). Additionally, the alcohol dose (g/kg/24 h) on the last day of training had a non-significant low positive correlation with the number of nosepokes on WD1 (Fig. 5C, *r* = 0.4025, *P* = 0.0512) and significant low positive correlation with the number of nosepokes on WD10 (Fig. 5D, *r* = 0.4343, *P* = 0.034).

## DISCUSSION

Despite many addiction research data, the field is still missing an adequate treatment for alcohol addiction. The reason is the lack of understanding of what is happening in the brain during withdrawal from the drug. The vast majority of research on studying alcohol drinking behaviours that employed animal models have been done on single housed animals. For social species like rats and mice, social exclusion is a stressful factor (Voikar *et al*., 2005). We aimed to develop a model to study alcohol-seeking after withdrawal behaviour in group-housed female mice with minimal human handling.

In our studies, we used female mice only; however, we believe that sex is an important biological factor, particularly in addiction research (Becker *et al*., 2017). In general, the use of female mice in IntelliCage is recommended (Kiryk *et al*., 2020). Though it has been shown that female mice can establish social hierarchy (Williamson *et al*., 2019), we did not observe any deviation in mice’s behaviour which would have signs of social dominance. Some studies have used male mice or mixed male and female groups of mice in IntelliCage (Kiryk *et al*., 2020). However, it would require male littermates to be placed in groups. Our premises do not have local breeding for large quantities of wild-type mice. Ordering from commercial suppliers would require long transportation that reflects on mouse behaviour. The aggression rates among male mice differ between batches. In our study, we emphasize stress and the human handling free model of group-housed mice. Therefore, the use of female mice is the question of feasibility and practicability.

Studies on alcohol drinking behaviour in group-housed mice in automated cages focus on different addiction-like behaviours (Radwanska and Kaczmarek, 2012; Smutek *et al*., 2014). However, our studies are the first to explore cue-induced alcohol-seeking behaviour after withdrawal in female mice. To reflect alcohol-seeking behaviour (one aspect of craving) in the animal model, we used cue-induced alcohol-directed behaviour. Thus, the number of nosepokes represents how much mice ‘want’ (crave for) alcohol. In turn, the number of licks shows how much mice ‘like’ alcohol. Previously we have shown that introducing long-term conditioning leads to an increased alcohol-seeking after withdrawal (Koskela *et al*., 2018). However, we also found that few possible confounding factors during alcohol conditioning may affect the result. To overcome this, we modified the protocol. In our previous study, mice had a two-bottle choice (one side of the corner was for alcohol and the other side was for water) in one conditioned corner where only alcohol drinking was associated with the cue (green light). Thus, four corners in automated cages were similar. However, each mouse had access to liquids only in two of the four corners (Koskela *et al*., 2018). In the present study, we placed alcohol in two sides of three conditioned corners, and the fourth, non-conditioned corner, had water in both sides. Also, doors to water were opened all the time. It excluded the possibility of conditioning to the door’s opening by nosepoke. All mice had access to liquids on all sides in all corners.

A substantial amount of literature provides evidence that intermittent access to alcohol leads to increased alcohol consumption (Wise, 1973; Simms *et al*., 2008; Hwa *et al*., 2011; Sabino *et al*., 2013; Carnicella *et al*., 2014). However, these studies do not perform extinction tests after withdrawal. Therefore, the intermittent access to the increasing concentrations of alcohol was switched to the continued access assuming that intermittency in our settings could result in extinction. For the sake of similar alcohol drinking training duration, we gave alcohol of the same concentration for 2 days until 12% ethanol solution was reached. Notably, we have observed a similar behaviour trend during the alcohol drinking training period and extinction tests compared with our previous findings (Koskela *et al*., 2018). However, we have found striking differences in the number of licks and, therefore, the amount of alcohol consumed. But there is a steady increase in both licks and doses. Notwithstanding, the amount of alcohol consumed at the end of the training period was similar to the previous result.

Interestingly, in the present study, the alcohol group performed significantly more cue-induced nosepokes than the water group on both WD1 and WD10 during extinction tests. Previously, we found such behaviour only on WD10 (Koskela *et al*., 2018). Moreover, we did not observe a time-dependent increase in the cue-induced alcohol-seeking, the phenomenon known as incubation of drug craving after withdrawal (Grimm *et al*., 2001). Although the neural mechanism that drives the incubation of drug craving is still not well defined, studies show that environmental enrichment and social interaction decrease and could even eliminate the already developed incubation of craving (Chauvet *et al*., 2012; Venniro *et al*., 2019). IntelliCage with conditioning corners and large social groups can be considered environmental enrichment. It may be why in our settings, the mice do not develop incubation of craving for alcohol. Environmental enrichment from social interaction provides a fascinating study subject to the future.

It is known that environmental enrichment and social interaction lead to increased individual differences in explorative behaviour among mice with identical genetic backgrounds (Freund *et al*., 2013; Torquet *et al*., 2018). We have taken a closer look at mice’s individual behaviour during extinction tests in our models. In the present study, we have observed that 33% of the alcohol group of mice performed more nosepokes on WD10 than on WD1. The same analysis of previous data (Koskela *et al*., 2018) on the same WD showed that an increased number of nosepokes was performed by 28% of the alcohol group of mice. The result suggests that despite eliminating possible confounding factors, the number of animals that show alcohol-seeking behaviour is similar.

Finally, our present findings demonstrate a correlation between the number of nosepokes on the last training day and the number of nosepokes on WD1 and WD10. Also, we observed a correlation between the alcohol dose consumed on the last training day and the number of nosepokes on WD1 and WD10. It is tempting to hypothesize that the neuroadaptation process that mediates cue-induced alcohol-seeking behaviour underlies such behavioural responses.

In conclusion, this study extends our previous alcohol craving model in female mice. Here we analysed possible confounding factors. Findings of the present study support the idea that long-term conditioning coupling with reward drug will result in cue-induced alcohol-seeking behaviour in group-housed mice. Finally, we address the individual behaviour of genetically identical mice living long-term in the group in environmental enrichment.

## Supplementary Material

Koskela_Alcohol_craving_mouse_model_Sup_Fig1_agab032

Koskela_Alcohol_craving_mouse_model_Sup_Fig2_agab032
