# Supplementary figures and images for: Female C57BL/6J Mice Show Alcohol-Seeking Behaviour after Withdrawal from Prolonged Alcohol Consumption in the Social Environment
Source: Alcohol Alcohol. 2021 Apr 24;57(4):405–12. doi: 10.1093/alcalc/agab032 (PMC9270993; doi:10.1093/alcalc/agab032)

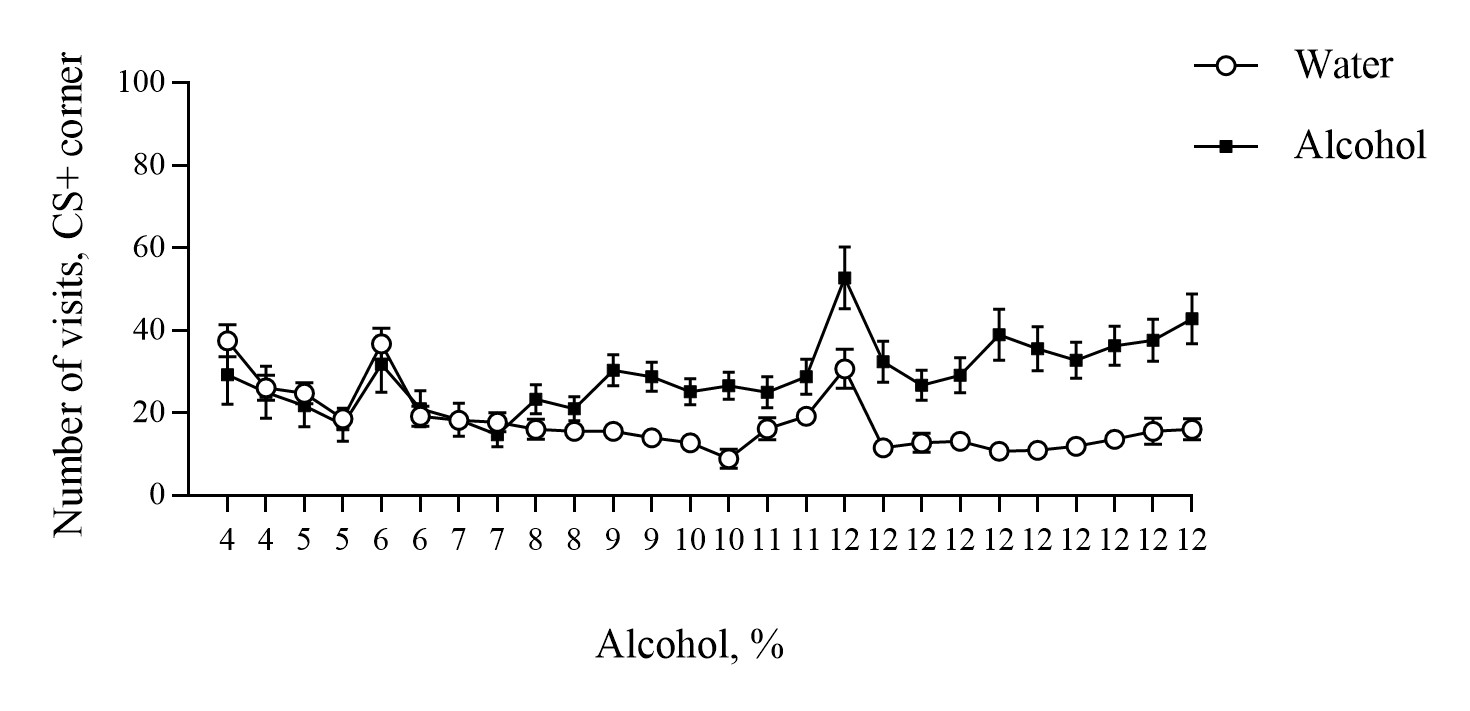

Supplement: Koskela_Alcohol_craving_mouse_model_Sup_Fig1_agab032 [file koskela_alcohol_craving_mouse_model_sup_fig1_agab032.jpeg]

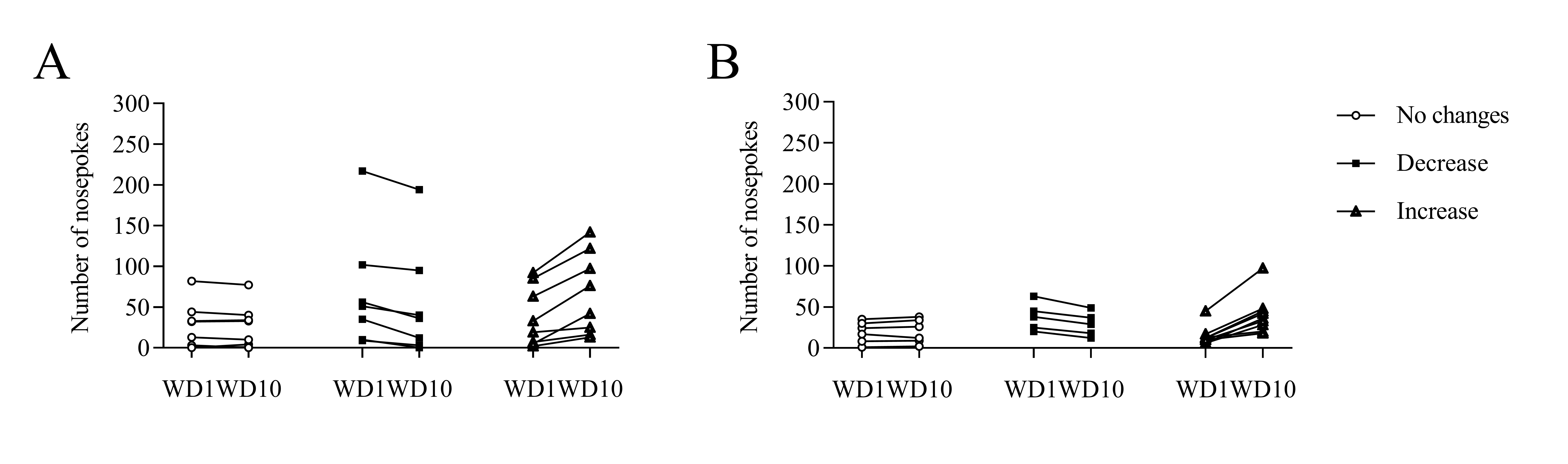

Supplement: Koskela_Alcohol_craving_mouse_model_Sup_Fig2_agab032 [file koskela_alcohol_craving_mouse_model_sup_fig2_agab032.jpeg]
